# Supplementary material for: Baseline Characterization of the Gut Microbiota of Field and Colony Populations of Phlebotomus tobbi and Preliminary Assessment of the Anti-Leishmanial Activity of Cultivable Bacteria
Source: Pathogens. 2026 Jun 23;15(7):658. doi: 10.3390/pathogens15070658 (PMC13416292; doi:10.3390/pathogens15070658)
Supplement: Supplementary file 1 [file pathogens-15-00658-s001.zip › pathogens-4357168-File S1/Heatmap/6.genus/heatmap_without_sample_clustering.pdf]

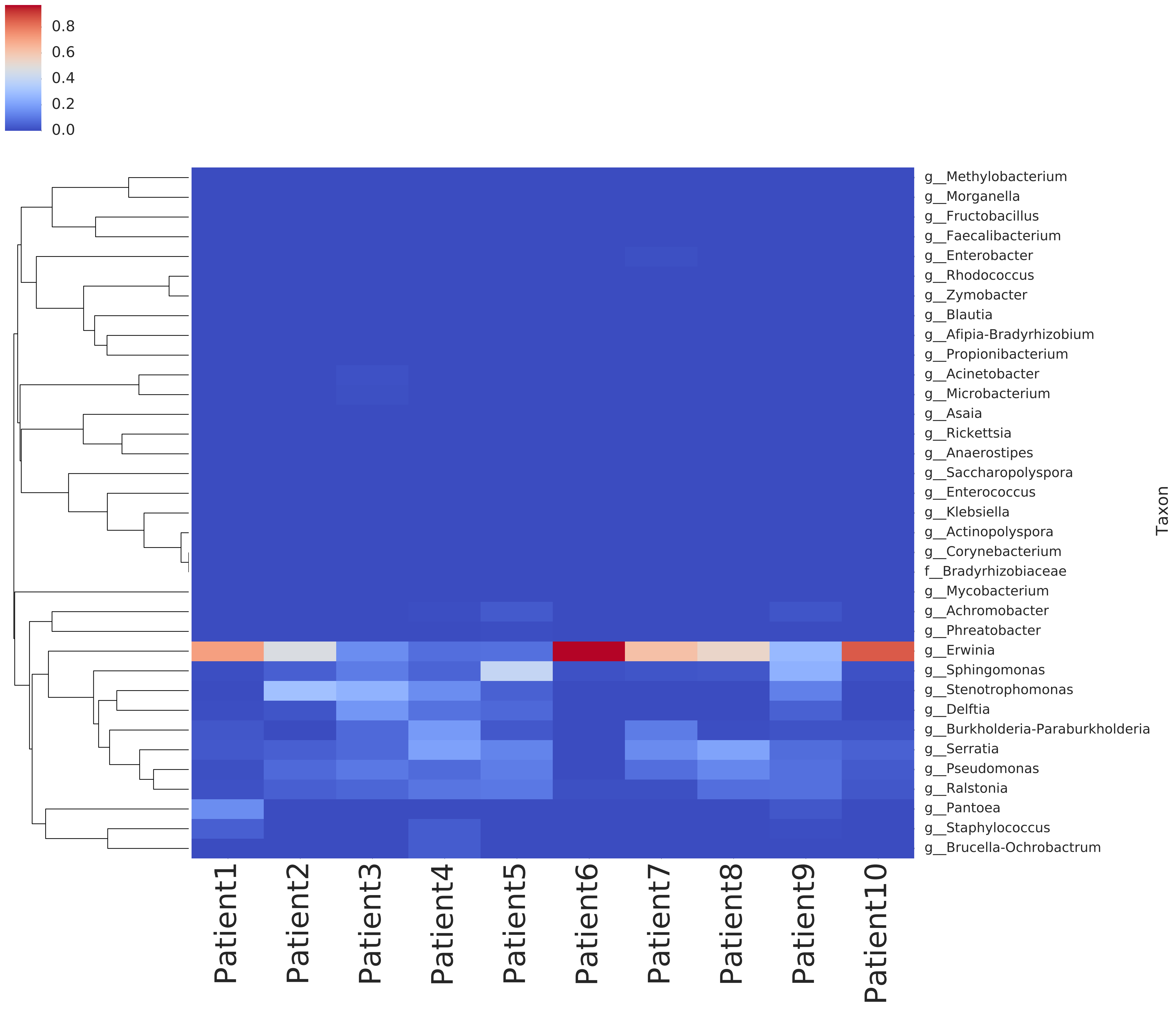
0.8
0.6
0.4
0.2
0.0
g__Methylobacterium
g__Morganella
g__Fructobacillus
g__Faecalibacterium
g__Enterobacter
g__Rhodococcus
g__Zymobacter
g__Blautia
g__Afipia-Bradyrhizobium
g__Propionibacterium
g__Acinetobacter
g__Microbacterium
g__Asaia
g__Rickettsia
g__Anaerostipes
g__Saccharopolyspora
g__Enterococcus
g__Klebsiella
g__Actinopolyspora
g__Corynebacterium
f__Bradyrhizobiaceae
g__Mycobacterium
g__Achromobacter
g__Phreatobacter
g__Erwinia
g__Sphingomonas
g__Stenotrophomonas
g__Delftia
g__Burkholderia-Paraburkholderia
g__Serratia
g__Pseudomonas
g__Ralstonia
g__Pantoea
g__Staphylococcus
g__Brucella-Ochrobactrum
Taxon
Patient1
Patient2
Patient3
Patient4
Patient5
Patient6
Patient7
Patient8
Patient9
Patient10
